# L-SHADE-Optimized Active Disturbance Rejection for Sensorless PMSM Drives Under Complex Uncertainties

**DOI:** 10.3390/s26113389

**Published:** 2026-05-27

**Authors:** Xiaoqing Chen, Tao Yang, Bowen Zhang, Ling Zhang

**Affiliations:** 1 School of Electrical Engineering, Chongqing Industry Polytechnic University, Chongqing 401120, China; yangtao@cqipc.edu.cn (T.Y.); zhangling@cqipu.edu.cn (L.Z.); 2 Chongqing Rail Transit Design and Research Institute Co., Ltd., Chongqing 401122, China; z632729573@163.com

**Keywords:** adaptive observer, L-SHADE, permanent magnet synchronous motor, robust sensorless control, variable-bandwidth LADRC-PLL

## Abstract

Sensorless permanent magnet synchronous motor (PMSM) drives rely on accurate rotor electrical angle and speed estimation, vulnerable to noisy currents, quantization, and sensor biases. Fixed-bandwidth phase-locked loops (PLLs) entail an intrinsic trade-off between fast transient tracking and high-frequency noise rejection. This paper proposes an adaptive PLL based on linear active disturbance rejection control (LADRC), where a virtual coordinate formulation treats electrical-angle mismatch as a lumped disturbance estimated online by a linear extended state observer (LESO). The observer bandwidth dynamically adapts to the LESO innovation. To optimize performance, adaptive-law parameters are tuned offline via success-history adaptive differential evolution with linear population size reduction (L-SHADE). Comparative simulations against a proportional-integral PLL indicate substantially improved robustness to measurement noise, analog-to-digital quantization, and current-sensor DC offset. Specifically, the speed root-mean-square error decreases from 68.9r/min to 20.7r/min under 0.15A additive noise, and from 1.55r/min to 0.48r/min under 12-bit quantization at 200r/min. These enhancements reduce reliance on high-precision sensing hardware, offering a practical solution for low-cost, highly reliable motor control in complex industrial environments.

## 1. Introduction

Permanent-magnet synchronous motors (PMSMs) are widely used in high-performance industrial drives due to their high power density, fast dynamic response, and high efficiency [1,2]. In many applications, sensorless control is preferred to avoid the cost, size constraints, wiring complexity, and reliability issues associated with rotor-position and speed sensors [3,4]. However, sensorless operation imposes stringent requirements on the accuracy and robustness of online estimation for the rotor electrical angle and speed [5], because the position estimate is directly involved in coordinate transformations and feedforward compensation, which determine the quality of current regulation and the smoothness of torque [6].

In a typical framework of indirect sensorless vector control, the rotor electrical angle is reconstructed from measured electrical variables using observer-based methods [7,8,9]. In practice, the observation process suffers from unavoidable imperfections in sensors and implementation, such as measurement noise, quantization errors of the ADC, and DC offsets of the current sensor [10,11,12]. These perturbations particularly affect estimators based on the PLL, because the correction dynamics of the PLL may amplify high-frequency components and lead to additional steady-state oscillations [13]. The design of the observer and the PLL must therefore balance fast transient tracking with the effective attenuation of high-frequency noise [14].

A conventional solution is to employ fixed-bandwidth tuning for linear or nonlinear observers and PLLs, where parameters are selected a priori according to nominal operating conditions [15,16]. While tuning with fixed parameters can yield good performance at a target point, it is prone to degradation when the operating point changes or when the disturbance statistics deviate from nominal assumptions [17,18]. Recent PMSM studies have attempted to go beyond fixed-parameter designs by introducing prescribed-performance observer-based speed control with data-driven RBF neural networks [19,20,21,22], differential neural-network learning and current prediction [23], decoupling sliding-mode observers for sensorless operation of bearingless permanent magnet vernier motors [24], virtual-flux-signal-injection hybrid active flux observers for zero-speed sensorless drive [25], and resonant ESO-based suppression of sampling-delay error under limited current sensing [26]. These results demonstrate important progress in robustness enhancement, yet they also reveal a common limitation: the improved performance is often obtained through additional learning modules, special signal injection, or highly task-specific observer structures, which can increase implementation complexity and reduce generality. Beyond sensorless control itself, related studies on prescribed-time synchronization and surrogate-model-assisted optimization further illustrate the growing interest in time-constrained control and efficient parameter search [27,28], while observer-based fuzzy adaptive control in other uncertain systems also confirms the appeal of adaptive disturbance compensation [29]. However, these methods do not directly resolve the central bandwidth trade-off in practical PMSM sensorless drives, namely the conflict between fast disturbance accommodation and noise amplification under measurement nonidealities. This inherent trade-off motivates the development of adaptive mechanisms with parameters optimized for robust behavior across a range of operating conditions and sensing nonidealities. In the revised discussion, the acronyms L-SHADE, LADRC, and LESO are repeated in the Introduction to improve readability and avoid ambiguity for readers who may not revisit the abstract.

To address these issues, this paper proposes an adaptive PLL based on LADRC and optimized by L-SHADE [30,31] for sensorless PMSM drives. The modeling is established in the *d*-*q* reference frame with a virtual coordinate (γ−δ) system that captures the mismatch in the estimation of the electrical angle as a lumped disturbance [32]. Based on this formulation, LESO is designed to estimate and compensate for the total disturbance online [8,15]. To resolve the conflict between fast tracking and noise suppression caused by a fixed observer bandwidth, the bandwidth of the LESO is adjusted online according to an error-dependent rule [9,32]. The parameters of the adaptive law are further optimized offline using L-SHADE under a multi-objective evaluation criterion [30,31,32].

This work introduces a virtual coordinate formulation to model the mismatch of the electrical angle in sensorless PMSM drives, expressing the resulting mismatch as a total disturbance suitable for formulation within the active disturbance rejection control (ADRC) framework. To estimate and reject this lumped disturbance online, an adaptive LADRC-PLL structure based on LESO is developed, which improves both transient tracking and steady-state accuracy. To overcome the limitations of fixed-bandwidth tuning, an online adaptive mechanism for the observer bandwidth is proposed to dynamically balance fast disturbance accommodation and the suppression of high-frequency noise. Additionally, an offline optimization procedure using L-SHADE with a multi-objective fitness function is employed to obtain robust parameters for the adaptive law under measurement noise, ADC quantization, and DC offsets of the sensor. The remainder of the paper details the electrical model and disturbance formulation (Section 2), the adaptive LADRC-PLL design (Section 3), simulation results (Section 4), and concluding remarks (Section 5).

## 2. System Modeling and Principle of Sensorless Control

This section establishes the electrical model of a PMSM in the *d*-*q* reference frame. It then introduces a virtual (γ−δ) coordinate system to represent the transformation errors caused by sensorless electrical angle estimation. The resulting model is rearranged into a total-disturbance form that is convenient for ADRC.

### 2.1. PMSM Mathematical Model in the d>-q Axes

Consider a PMSM expressed in a rotor-oriented rotating reference frame. The stator currents are represented by $\left(i_{d},i_{q}\right)$, while $v_{d}$ and $v_{q}$ denote the corresponding *d*- and *q*-axis stator voltages. The associated stator flux linkages are $\left(\psi_{d},\psi_{q}\right)$. The electrical angular speed is $\omega_{e}=\frac{d\theta_{e}}{dt}$, where $\theta_{e}$ is the electrical rotor angle. The motor parameters include the stator resistance $R_{s}$, the *d*- and *q*-axis inductances $L_{d}$ and $L_{q}$, and the permanent-magnet flux linkage $\psi_{f}$.

#### 2.1.1. Flux Linkage Relations

For a standard PMSM, the flux linkage relations are written as

(1) $$\psi_{d}=L_{d}i_{d}+\psi_{f},\;\psi_{q}=L_{q}i_{q}.$$

The permanent-magnet flux linkage $\psi_{f}$ is assumed to be aligned with the *d*-axis. For a surface-mounted PMSM (SPMSM), the inductance asymmetry is negligible and one may set $L_{d}=L_{q}=L_{s}$.

#### 2.1.2. Voltage Equations

The stator voltage equations in the *d*-*q* frame are given by [33].

(2) $$\begin{array}{cc} v_{d} & =R_{s}i_{d}+\frac{d\psi_{d}}{dt}-\omega_{e}\psi_{q},\\ v_{q} & =R_{s}i_{q}+\frac{d\psi_{q}}{dt}+\omega_{e}\psi_{d}. \end{array}$$

Substituting $\psi_{d}=L_{d}i_{d}+\psi_{f}$ and $\psi_{q}=L_{q}i_{q}$, and using the assumption that $\psi_{f}$ is constant in the rotor frame, the voltage equations can be expanded to

(3) $$\begin{array}{cc} v_{d} & =R_{s}i_{d}+L_{d}\frac{di_{d}}{dt}-\omega_{e}L_{q}i_{q},\\ v_{q} & =R_{s}i_{q}+L_{q}\frac{di_{q}}{dt}+\omega_{e}\left(L_{d}i_{d}+\psi_{f}\right). \end{array}$$

In these expressions, the coupling terms $-\omega_{e}\psi_{q}$ and $+\omega_{e}\psi_{d}$ represent the back-electromotive-force induced by the rotating reference frame, while $R_{s}i_{d}$ and $R_{s}i_{q}$ denote the copper voltage drops.

### 2.2. Virtual Coordinate System Formulation and Total Disturbance with Angle Error

In sensorless PMSM drives, the rotor electrical angle used for the coordinate transformation is not measured directly but estimated as ${\hat{\theta }}_{e}$. Consequently, the transformation frame is misaligned with respect to the true rotor-oriented *d*-*q* frame. To explicitly capture the effect of this misalignment, a virtual (γ−δ) coordinate system is introduced.

The electrical angle estimation error is defined as

(4) $$\Delta \theta_{e}={\hat{\theta }}_{e}-\theta_{e}.$$

**Figure 1:**
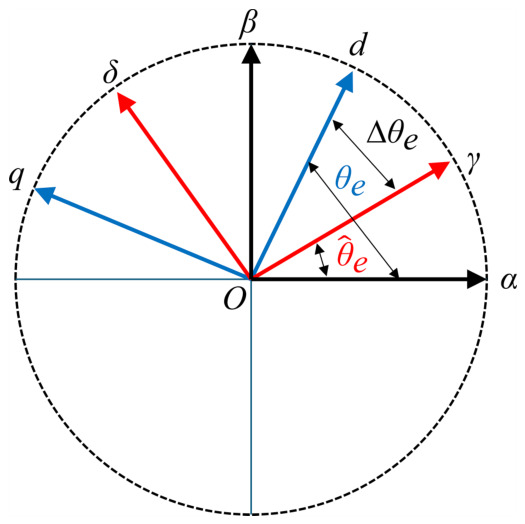
The virtual (γ−δ) coordinate system used in sensorless PMSM control. The virtual frame is rotated relative to the true (d−q) frame by the electrical angle error $\Delta \theta_{e}$.

The virtual-frame currents are obtained from the true *d*-*q* currents through the rotation induced by $\Delta \theta_{e}$, as illustrated in Figure 1 [34].

(5) $$\left[\begin{array}{c} i_{\gamma }\\ i_{\delta } \end{array}\right]=\left[\begin{array}{cc} \cos\Delta \theta_{e} & \sin\Delta \theta_{e}\\ -\sin\Delta \theta_{e} & \cos\Delta \theta_{e} \end{array}\right]\left[\begin{array}{c} i_{d}\\ i_{q} \end{array}\right].$$

To avoid ambiguity in the subsequent derivations, the main symbols used in Section 2 and Section 3 are summarized here: $i_{d}$, $i_{q}$, $i_{\gamma }$, and $i_{\delta }$ denote the stator-current components in the true and virtual rotating frames, respectively; $v_{d}$, $v_{q}$, $v_{\gamma }$, and $v_{\delta }$ denote the corresponding stator-voltage components; $\theta_{e}$ and ${\hat{\theta }}_{e}$ are the actual and estimated electrical rotor angles; $\Delta \theta_{e}$ is the electrical angle estimation error; $\omega_{e}$ and ${\hat{\omega }}_{e}$ are the actual and estimated electrical angular speeds; $R_{s}$ is the stator resistance; $L_{d}$, $L_{q}$, and $L_{s}$ are the *d*-axis, *q*-axis, and equivalent stator inductances; $\psi_{d}$ and $\psi_{q}$ are the stator flux linkages; $\psi_{f}$ is the permanent-magnet flux linkage; and $d_{\gamma }\left(t\right)$, $d_{\delta }\left(t\right)$, $d_{\gamma }^{\theta }\left(t\right)$, and $d_{\delta }^{\theta }\left(t\right)$ denote the lumped disturbances in the virtual frame.

For dimensional consistency, all terms in the current-dynamic equations are expressed in units of current derivative (A/s). Specifically, $R_{s}i/L_{s}$, $v/L_{s}$, ωi, and $\omega \psi_{f}/L_{s}$ all have units of A/s, while the lumped disturbances $d_{\gamma }\left(t\right)$ and $d_{\delta }\left(t\right)$ are defined with the same unit. This clarification has been used consistently in the revised model equations below. The state-space representation and disturbance interpretation are consistent with the extended-EMF modeling framework.

#### Total Disturbance Induced by Angle Error

For an explicit disturbance expression, the surface-mounted PMSM case is considered in this subsection, i.e., $L_{d}=L_{q}=L_{s}$ [35]. In the true *d*-*q* frame, the current dynamics are

(6) $$\begin{array}{cc} {\dot{i}}_{d} & =-\frac{R_{s}}{L_{s}}i_{d}+\frac{1}{L_{s}}v_{d}+\omega_{e}i_{q},\\ {\dot{i}}_{q} & =-\frac{R_{s}}{L_{s}}i_{q}+\frac{1}{L_{s}}v_{q}-\omega_{e}i_{d}-\frac{\omega_{e}\psi_{f}}{L_{s}}. \end{array}$$

where ${\dot{i}}_{d}$ and ${\dot{i}}_{q}$ denote the time derivatives of the currents and therefore have units of A/s. Accordingly, the terms $\frac{R_{s}}{L_{s}}i_{d}$, $\frac{R_{s}}{L_{s}}i_{q}$, $\frac{1}{L_{s}}v_{d}$, $\frac{1}{L_{s}}v_{q}$, $\omega_{e}i_{q}$, $\omega_{e}i_{d}$, and $\frac{\omega_{e}\psi_{f}}{L_{s}}$ are dimensionally consistent with A/s.

When the coordinate transformation is driven by ${\hat{\theta }}_{e}$, the magnet-induced term that is originally present only in the *q*-axis equation appears as an angle-dependent contribution in the virtual (γ−δ) dynamics. Specifically, the permanent-magnet forcing term can be decomposed in the virtual frame as

(7) $$-\frac{\omega_{e}\psi_{f}}{L_{s}}\left[\begin{array}{c} \sin\Delta \theta_{e}\\ \cos\Delta \theta_{e} \end{array}\right].$$

By contrast, the nominal model used in the controller typically assigns the known magnet coupling only to the δ-axis using the estimated speed ${\hat{\omega }}_{e}$. This yields the following virtual-frame structure:

(8) $$\begin{array}{cc} {\dot{i}}_{\gamma } & =-\frac{R_{s}}{L_{s}}i_{\gamma }+\frac{1}{L_{s}}v_{\gamma }+{\hat{\omega }}_{e}i_{\delta }+d_{\gamma }\left(t\right),\\ {\dot{i}}_{\delta } & =-\frac{R_{s}}{L_{s}}i_{\delta }+\frac{1}{L_{s}}v_{\delta }-{\hat{\omega }}_{e}i_{\gamma }-\frac{{\hat{\omega }}_{e}\psi_{f}}{L_{s}}+d_{\delta }\left(t\right). \end{array}$$

Here, $d_{\gamma }\left(t\right)$ and $d_{\delta }\left(t\right)$ are defined as acceleration-like current disturbances with units of A/s, rather than current quantities in A. This definition resolves the possible dimensional ambiguity: every term on the right-hand side of the two equations has the same unit as ${\dot{i}}_{\gamma }$ and ${\dot{i}}_{\delta }$.

The angle-error-induced disturbance terms are therefore

(9) $$\begin{array}{cc} d_{\gamma }^{\theta }\left(t\right) & =-\frac{\psi_{f}}{L_{s}}\;\omega_{e}\sin\Delta \theta_{e},\\ d_{\delta }^{\theta }\left(t\right) & =\frac{\psi_{f}}{L_{s}}\left({\hat{\omega }}_{e}-\omega_{e}\cos\Delta \theta_{e}\right). \end{array}$$

In ADRC modeling, the total disturbance is typically defined as the sum of the angle-error-induced term and an additional lumped uncertainty:

(10) $$\begin{array}{cc} d_{\gamma }\left(t\right) & =d_{\gamma }^{\theta }\left(t\right)+d_{\mathrm{unc},\gamma }\left(t\right),\\ d_{\delta }\left(t\right) & =d_{\delta }^{\theta }\left(t\right)+d_{\mathrm{unc},\delta }\left(t\right). \end{array}$$

Finally, since $\Delta \theta_{e}={\hat{\theta }}_{e}-\theta_{e}$, the speed mismatch satisfies $\omega_{e}={\hat{\omega }}_{e}-\Delta {\dot{\theta }}_{e}$, which gives an alternative disturbance expression:

(11) $$\begin{array}{cc} d_{\gamma }^{\theta }\left(t\right) & =-\frac{\psi_{f}}{L_{s}}\left({\hat{\omega }}_{e}-\Delta {\dot{\theta }}_{e}\right)\sin\Delta \theta_{e},\\ d_{\delta }^{\theta }\left(t\right) & =\frac{\psi_{f}}{L_{s}}\left[{\hat{\omega }}_{e}-\left({\hat{\omega }}_{e}-\Delta {\dot{\theta }}_{e}\right)\cos\Delta \theta_{e}\right]. \end{array}$$

This formulation shows that the electrical angle estimation error introduces axis coupling through $\sin\Delta \theta_{e}$ and modifies the magnet contribution through $\cos\Delta \theta_{e}$. As a result, the mismatch between the nominal and the actual virtual-frame dynamics can be treated as a lumped total disturbance for robust sensorless control design.

## 3. L-SHADE Optimized Adaptive LADRC-PLL Design

This section presents the proposed sensorless control scheme based on LADRC-PLL. The key idea is to use LESO to estimate the lumped disturbance caused by model uncertainties and electrical angle mismatch, and resolve the inherent bandwidth tuning conflict through an online adaptive bandwidth mechanism. The adaptive law parameters are subsequently optimized offline using L-SHADE to maintain robust performance under nonideal conditions.

### 3.1. LESO Structure and the Fixed-Bandwidth Conflict

#### 3.1.1. Standard Second-Order LESO

Consider a scalar channel that can be written in the ADRC canonical form [36].

(12) $${\dot{x}}_{1}=x_{2},\;{\dot{x}}_{2}=f\left(t\right)+b_{0}u,\;y=x_{1},$$

where $x_{1}$ is the measured output, $x_{2}$ is its derivative, *u* is the control input, $b_{0}$ is the nominal input gain, and f(t) denotes the total disturbance that lumps unknown dynamics, parameter variations, and exogenous perturbations.

The second-order LESO augments f(t) as an extended state and estimates $\left(x_{1},f\right)$ as [37].

(13) $$\begin{array}{cc} {\dot{z}}_{1} & =z_{2}+\beta_{1}\left(y-z_{1}\right),\\ {\dot{z}}_{2} & =b_{0}u+\beta_{2}\left(y-z_{1}\right), \end{array}$$

where $z_{1}$ estimates $x_{1}$ and $z_{2}$ estimates f(t).

With the bandwidth parameterization, the observer gains are commonly selected as:

(14) $$\beta_{1}=2\omega_{o},\;\beta_{2}=\omega_{o}^{2},$$

where $\omega_{o}>0$ is the observer bandwidth.

#### 3.1.2. Standard Third-Order LESO

For a second-order plant with a time-varying disturbance whose derivative is non-negligible, a third-order LESO is often used [38]:

(15) $$\begin{array}{cc} {\dot{z}}_{1} & =z_{2}+\beta_{1}\left(y-z_{1}\right),\\ {\dot{z}}_{2} & =z_{3}+b_{0}u+\beta_{2}\left(y-z_{1}\right),\\ {\dot{z}}_{3} & =\beta_{3}\left(y-z_{1}\right), \end{array}$$

where $z_{1}$ estimates $x_{1}$, $z_{2}$ estimates ${\dot{x}}_{1}$, and $z_{3}$ estimates the total disturbance f(t).

Using the bandwidth-based tuning, the gains are chosen as [3].

(16) $$\beta_{1}=3\omega_{o},\;\beta_{2}=3\omega_{o}^{2},\;\beta_{3}=\omega_{o}^{3}.$$

In the proposed LADRC-PLL, the lumped disturbance is treated as time-varying; therefore, the third-order LESO above is adopted for implementation, and the adaptive bandwidth law in Section 3.2 updates $\omega_{o}\left(t\right)$ online while retaining this third-order gain structure.

#### 3.1.3. Fixed Bandwidth: Fast Tracking Versus High-Frequency Noise Rejection

In LADRC-PLL, the LESO provides real-time estimates of the extended state (i.e., the lumped disturbance) that includes the electrical angle mismatch effects derived in Section 2. The bandwidth $\omega_{o}$ plays a dual role. A larger $\omega_{o}$ increases the observer gains $\left\{\beta_{i}\right\}$ and accelerates the convergence of the estimation error, improving transient tracking during rapid speed changes, load disturbances, and strong model mismatch. However, increasing $\omega_{o}$ also amplifies measurement noise and quantization ripple because the correction term $\beta_{i}\left(y-z_{1}\right)$ injects high-frequency components into the observer states. Consequently, the estimated disturbance and the PLL input become noisier, which degrades steady-state angle accuracy and may excite high-frequency oscillations.

This leads to an intrinsic tuning conflict: a single fixed $\omega_{o}$ cannot simultaneously guarantee aggressive transient tracking and strong high-frequency noise suppression. In practice, selecting a large $\omega_{o}$ yields fast dynamic response but poor noise robustness, whereas a small $\omega_{o}$ improves noise immunity but slows down the observer response. This conflict motivates an online adaptive bandwidth mechanism.

### 3.2. Online Adaptive Bandwidth Mechanism

To address the fixed-bandwidth conflict, the observer bandwidth is adjusted online according to an instantaneous error indicator. Let

(17) $$e_{\mathrm{obs}}\left(t\right)=y\left(t\right)-z_{1}\left(t\right),$$

denote the LESO innovation (output estimation error). Intuitively, a large $|e_{\mathrm{obs}}|$ indicates either a rapid transient or a substantial mismatch, where a larger $\omega_{o}$ is desired; conversely, a small $|e_{\mathrm{obs}}|$ corresponds to quasi-steady operation, where a smaller $\omega_{o}$ is preferred to attenuate measurement noise.

#### Error-Dependent Bandwidth Law

An exponential mapping provides a smooth and bounded adjustment rule:

(18) $$\omega_{o}^{⋆}\left(t\right)=\omega_{\min}+\left(\omega_{\max}-\omega_{\min}\right)\left(1-\exp\left(-k_{\omega }\left|e_{\mathrm{obs}}\left(t\right)\right|\right)\right),$$

where $\omega_{\min}>0$ and $\omega_{\max}>\omega_{\min}$ are the minimum and maximum observer bandwidths, and $k_{\omega }>0$ controls the sensitivity to the error magnitude. This law satisfies $\omega_{o}^{⋆}\to \omega_{\min}$ as $|e_{\mathrm{obs}}|\to 0$ and $\omega_{o}^{⋆}\to \omega_{\max}$ as $|e_{\mathrm{obs}}|\to \infty$.

To avoid abrupt gain variation and improve numerical stability, the implemented bandwidth is further filtered by a first-order dynamics:

(19) $${\dot{\omega }}_{o}\left(t\right)=\frac{1}{\tau_{\omega }}\left(\omega_{o}^{⋆}\left(t\right)-\omega_{o}\left(t\right)\right),$$

where $\tau_{\omega }>0$ is a smoothing time constant. The observer gains are then updated online via the standard bandwidth parameterization. For the third-order LESO, this yields:

(20) $$\beta_{1}\left(t\right)=3\omega_{o}\left(t\right),\;\beta_{2}\left(t\right)=3\omega_{o}^{2}\left(t\right),\;\beta_{3}\left(t\right)=\omega_{o}^{3}\left(t\right).$$

With this mechanism, the observer becomes aggressive when the estimation residual increases, enabling rapid disturbance accommodation. Conversely, it automatically reduces the bandwidth near steady state to suppress high-frequency noise, ADC quantization ripple, and sensor bias-induced fluctuations.

### 3.3. Offline L-SHADE Optimization of Adaptive Law Parameters

Although the adaptive bandwidth law introduces only a few intuitive parameters (e.g., $\omega_{\min}$, $\omega_{\max}$, $k_{\omega }$, and $\tau_{\omega }$), their coupled impact on transient and steady-state performance is highly nonlinear and depends heavily on operating conditions and sensing nonidealities. To obtain a robust parameter set, an offline optimization is conducted using L-SHADE.

#### 3.3.1. Optimization Variables and Evaluation Protocol

Let the design vector be

(21) $$p={\left[\begin{array}{cccc} \omega_{\min} & \omega_{\max} & k_{\omega } & \tau_{\omega } \end{array}\right]}^{T},$$

subject to the constraints $p\in [p_{\min},p_{\max}]$. For each candidate p, time-domain simulations are executed over a predefined scenario set encompassing speed transients, load steps, and measurement nonidealities. The performance indices are then computed from the resulting trajectories.

#### 3.3.2. L-SHADE Procedure

**Figure 2:**
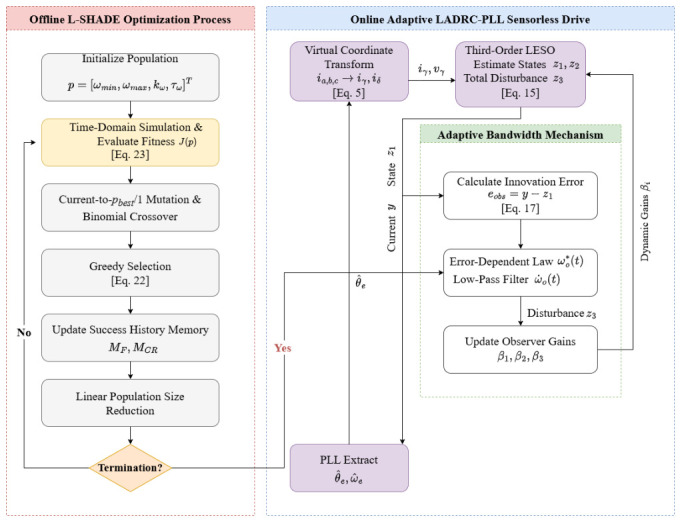
Implementation flowchart of the proposed adaptive LADRC-PLL optimized by L-SHADE.

L-SHADE is an advanced differential evolution (DE) variant that extends success-history adaptive DE with linear population size reduction [5]. As illustrated in the offline optimization process of the proposed flowchart (see Figure 2), the algorithm iteratively evolves a population of decision variables $p={\left[\omega_{min},\omega_{max},k_{\omega },\tau_{\omega }\right]}^{T}$ to minimize a composite fitness cost J(p).

The evolutionary process evaluates the fitness via time-domain simulation and consists of the following key stages per generation *G*:

- Mutation and Crossover: For each target vector $x_{i,G}$, a “current-to-*p*best/1” mutation strategy generates a mutant vector utilizing the current individual, a randomly selected top-performing individual (*p*best), and difference vectors from the population and an external archive. A binomial crossover is then applied to mix the mutant and target vectors, yielding a trial vector $u_{i,G}$.
- Greedy Selection: The survival of the trial vector is determined by evaluating the objective function:
  (22) $$x_{i,G+1}:=\left\{\begin{array}{cc} u_{i,G}, & \mathrm{if}\;J\left(u_{i,G}\right)\leq J\left(x_{i,G}\right),\\ x_{i,G}, & \mathrm{otherwise}. \end{array}\right.$$
  If the trial vector replaces the parent, the replaced parent is stored in a bounded archive A to maintain genetic diversity.
- Success History Update: Instead of using fixed parameters, L-SHADE dynamically adapts its crossover rate (CR) and mutation scale factor (*F*). These are sampled based on a success-history memory ($M_{CR}$ and $M_{F}$), which is continuously updated using the parameter values that successfully produced surviving trial vectors in recent generations.
- Linear Population Size Reduction (LPSR): To accelerate convergence, the population size $NP_{G}$ is linearly decreased from an initial size $NP_{\mathrm{init}}$ to a minimum size $NP_{\min}$ over the maximum number of generations.

To balance transient speed, overshoot, and estimation accuracy for the online adaptive LADRC-PLL drive, the fitness function evaluated during the simulation is defined as:

(23) $$J\left(p\right)=w_{1}\;{\bar{M}}_{p}\left(p\right)+w_{2}\;{\bar{T}}_{s}\left(p\right)+w_{3}\;{\bar{\mathrm{RMSE}}}_{\theta }\left(p\right)+w_{4}\;{\bar{\mathrm{RMSE}}}_{\omega }\left(p\right)$$

The four weights ($w_{1}$ to $w_{4}$) are normalized and selected to give a comparable contribution to each term, avoiding domination by any single metric. Specifically, $w_{1}$ and $w_{2}$ penalize the normalized peak overshoot (${\bar{M}}_{p}$) and settling time (${\bar{T}}_{s}$) to maintain transient quality. Meanwhile, $w_{3}$ and $w_{4}$ prioritize the normalized root-mean-square errors of the angle (${\bar{\mathrm{RMSE}}}_{\theta }$) and speed (${\bar{\mathrm{RMSE}}}_{\omega }$) estimates, which directly dictate coordinate transformation precision and PLL locking performance. Through this automated offline tuning, L-SHADE effectively identifies an optimal parameter vector p that guarantees precise state estimation and robust dynamic response under nonideal sensing conditions.

**Figure 3:**
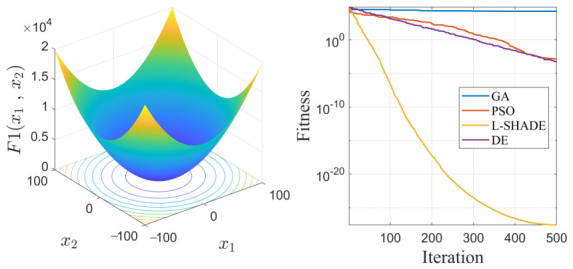
Convergence comparison of L-SHADE, GA, PSO, and DE on the benchmark function.

To validate the proposed strategy, L-SHADE is evaluated against standard algorithms (GA, PSO, and DE) using the benchmark function $F_{1}\left(x\right)=\sum x_{i}^{2}$, as shown in Figure 3. The convergence curves indicate that while GA converges prematurely and PSO and DE lack fine-tuning precision, L-SHADE converges rapidly and achieves an accuracy near $10^{-20}$. This search capability justifies using L-SHADE for offline tuning to obtain a parameter vector p that ensures robust motor drive stability.

## 4. Simulation Results and Performance Evaluation

This section evaluates the proposed L-SHADE-optimized adaptive-bandwidth LADRC-PLL through time-domain simulations. The emphasis is placed on nominal steady-state accuracy, transient tracking capability, and disturbance rejection, which together characterize the baseline performance prior to introducing severe sensing nonidealities.

### 4.1. Simulation Setup

**Figure 4:**
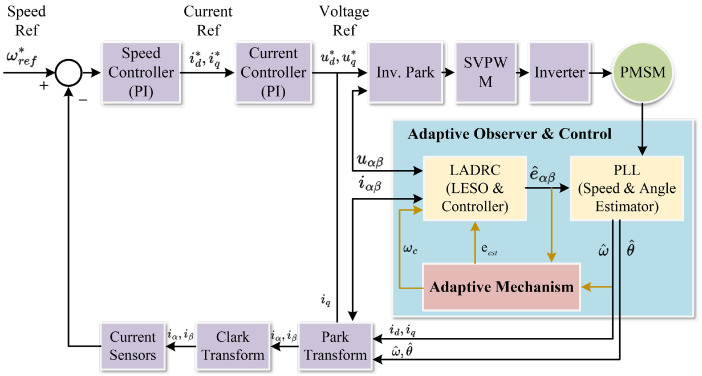
Proposed adaptive-bandwidth LADRC-PLL sensorless control scheme.

The PMSM drive system is simulated in MATLAB/Simulink (Version R2023b) in the synchronous reference frame under a sensorless control architecture, where the rotor electrical angle ${\hat{\theta }}_{e}$ and electrical speed ${\hat{\omega }}_{e}$ are provided by the proposed LADRC-PLL. To provide a clear baseline for the computational load of the proposed algorithm, all simulations were executed on a workstation equipped with an Intel Core i7-13700 @ 2.10 GHz processor, 16 GB of RAM, and a 64-bit Windows 11 operating system. The control structure follows the overall scheme in Figure 4, and the system model and the angle-error-induced disturbance formulation are consistent with Section 2. The main parameters of the simulated PMSM are listed in Table 1.

The offline L-SHADE optimization yielded the following adaptive-law parameters: $\omega_{\min}=80\;\mathrm{rad}/s$, $\omega_{\max}=300\;\mathrm{rad}/s$, $k_{\omega }=0.8$, and $\tau_{\omega }=5\;\mathrm{ms}$. These values were used throughout the simulations reported in this section.

To assess the nominal behavior, three representative operating conditions are evaluated. Initially, the drive operates in a steady state at a constant reference speed of $n^{⋆}=1200\;r/\min$ under a constant load torque, during which the three-phase currents, electromagnetic torque, electrical angle, and speed estimation are recorded. Subsequently, a speed command step is applied at t=0.6s to analyze the dynamic tracking performance and the convergence of the speed estimation error. Finally, a step load torque is injected at t=1.0s to quantify the instantaneous speed drop and the recovery time under abrupt torque disturbances.

Unless otherwise specified, the results in this section correspond to the nominal parameter set obtained by the offline L-SHADE tuning described in Section 3.3. The resulting adaptive-law parameters are reported explicitly below to facilitate reproducibility.

To compare computational load among the observer schemes, the average execution time of one sensorless observer update per control cycle was recorded under identical simulation settings (same fixed-step solver and profiling configuration). The measured values are 0.253μs for the PI-PLL, 0.506μs for the fixed-bandwidth LADRC-PLL, and 0.731μs for the proposed adaptive LADRC-PLL; offline L-SHADE tuning is excluded because it is executed only once before runtime.

### 4.2. Nominal Steady-State and Dynamic Performance

#### 4.2.1. Steady-State Performance at Rated Speed

**Figure 5:**
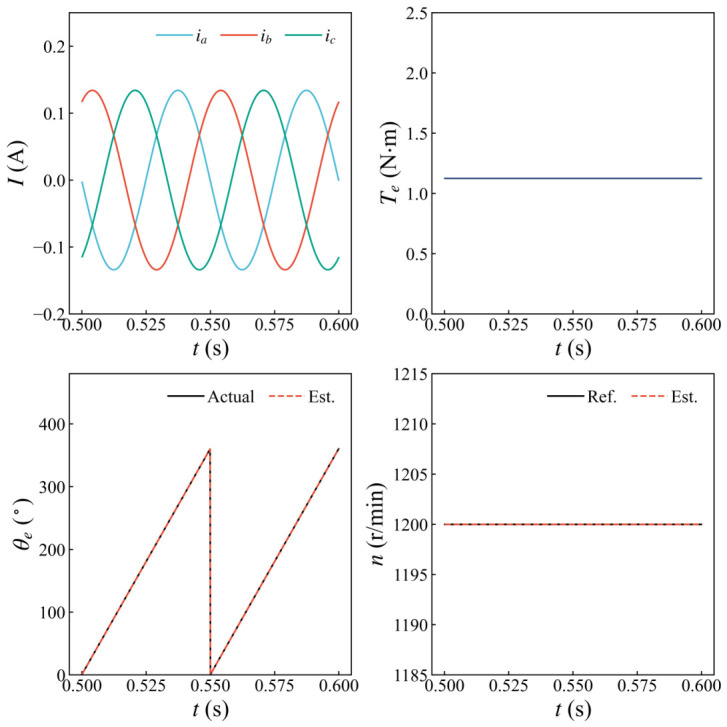
Nominal steady-state waveforms at n=1200r/min: stator currents, electromagnetic torque, electrical angle, and estimated speed.

As shown in Figure 5, under the steady-state condition at 1200r/min, the three-phase currents $i_{a}$, $i_{b}$, and $i_{c}$ remain well balanced with a peak amplitude of approximately 0.135A, exhibiting a highly sinusoidal profile that confirms the proper execution of the control algorithm and coordinate transformation. The electromagnetic torque $T_{e}$ stabilizes at approximately 1.13N·m with negligible steady-state ripple. This smooth torque output implies that the estimated electrical angle is highly accurate. Furthermore, the estimated electrical angle perfectly overlaps the actual angle trajectory, and the estimated speed tracks the reference of 1200r/min with no visible steady-state error. These quantitative observations substantiate the high steady-state estimation precision of the proposed LADRC-PLL.

#### 4.2.2. Dynamic Speed Tracking Under a Speed Step

**Figure 6:**
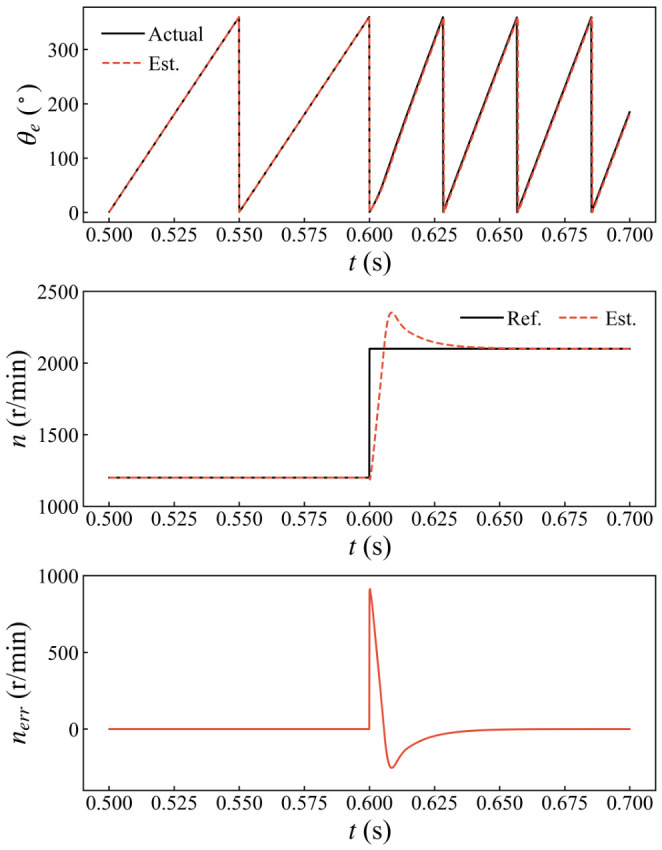
Speed step response at 0.6 s.

Figure 6 illustrates the transient response when the speed command steps from 1200r/min to 2100r/min at t=0.6s. The estimated speed rapidly tracks the new reference, accompanied by a transient overshoot peaking at approximately 2350r/min. Correspondingly, the speed tracking error $n_{\mathrm{err}}$ spikes to 900r/min at the moment of the step change, drops to a negative peak of roughly −250r/min, and subsequently converges to zero within a brief settling time of approximately 40ms (at t≈0.64s). Throughout this dynamic acceleration phase, the estimated electrical angle continues to perfectly coincide with the actual angle trajectory. This uninterrupted synchronization highlights the rapid disturbance accommodation capability of the adaptive-bandwidth LADRC-PLL, which ensures stable phase-locking and robust current regulation under severe speed variations.

#### 4.2.3. Load Disturbance Rejection Under a Step Torque

**Figure 7:**
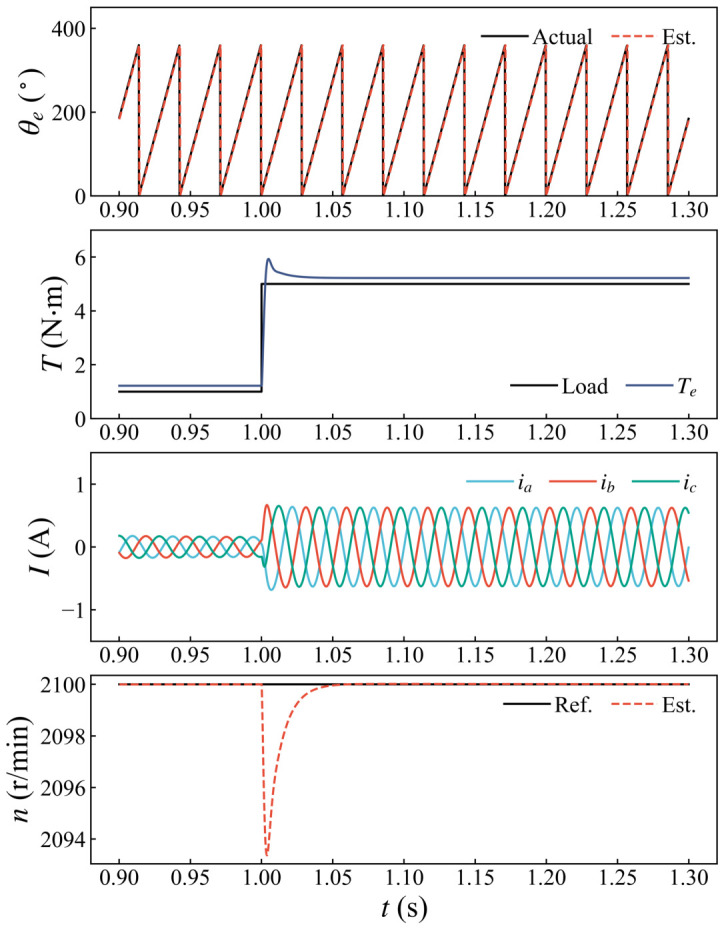
Load step response at t=1.0s: electrical angle, electromagnetic torque, phase currents, and estimated speed.

Figure 7 evaluates the system performance under a sudden load disturbance, where a step load torque from 1N·m to 5N·m is applied at t=1.0s while the motor operates at 2100r/min. Upon the load impact, the estimated speed experiences a minor transient drop of approximately 6.5r/min, reaching a minimum of 2093.5r/min. However, the speed quickly recovers to the reference value within a brief settling time of roughly 50ms (at t≈1.05s). Correspondingly, the electromagnetic torque $T_{e}$ exhibits a rapid dynamic response, peaking at approximately 6N·m before settling to balance the new load. This is accompanied by a smooth and proportional increase in the three-phase current amplitudes to roughly 0.65A. Throughout this abrupt mechanical transition, the estimated electrical angle maintains perfect alignment with the actual angle trajectory. These results confirm that the proposed adaptive-bandwidth LADRC-PLL effectively decouples load disturbances from the observer dynamics, ensuring stable phase-locking and high-fidelity sensorless operation under severe load variations.

### 4.3. Robustness Analysis

#### 4.3.1. Robustness Against Measurement Noise

**Figure 8:**
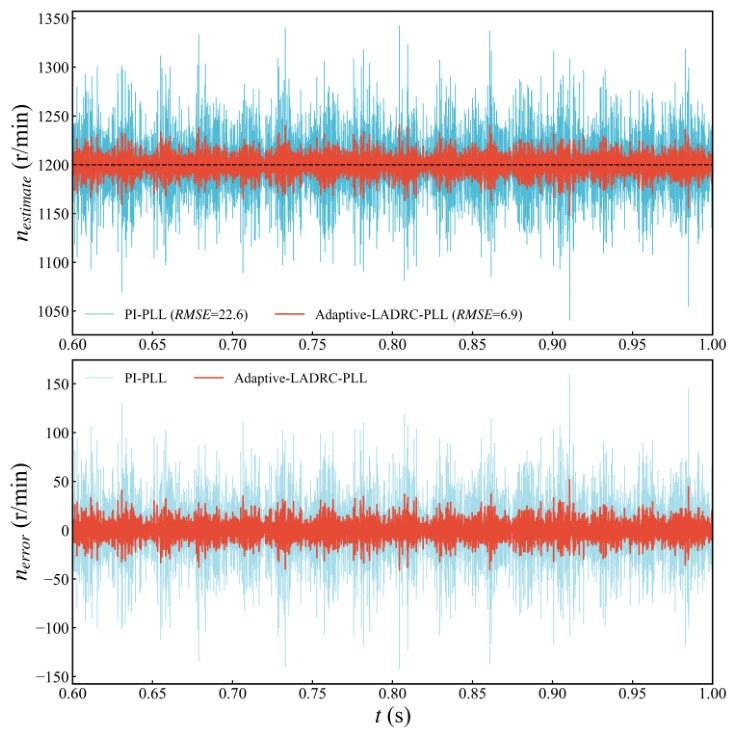
Speed estimation under 0.05A measurement noise.

**Figure 9:**
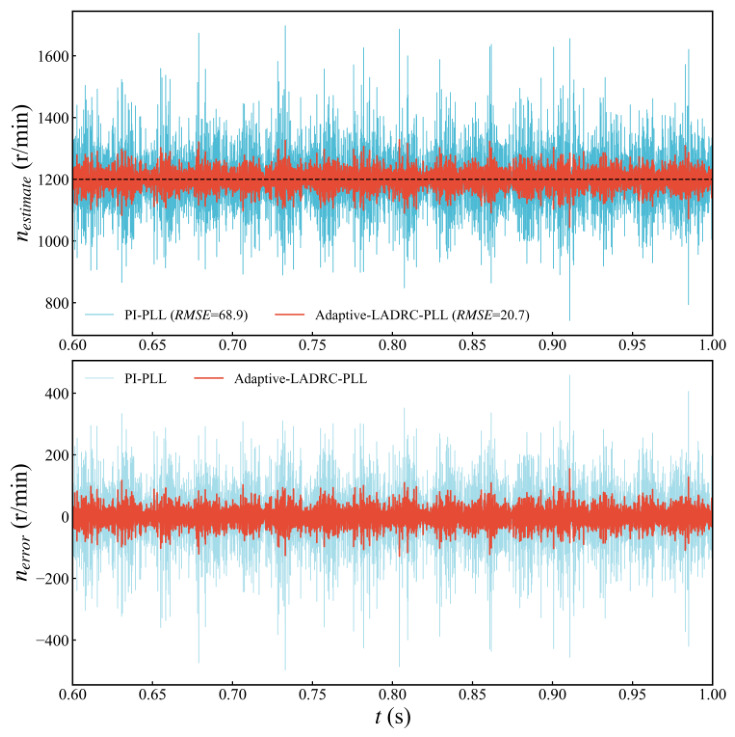
Speed estimation under 0.15A measurement noise.

To evaluate the robustness against measurement noise, additive noise is injected into the sampled phase currents, and the speed estimation performance of the proposed LADRC-PLL is compared with that of a conventional PI-PLL. Two representative noise intensities are considered: a light noise level with an amplitude of 0.05A (Figure 8) and a severe noise level of 0.15A (Figure 9).

Under the 0.05A noise condition, the PI-PLL exhibits noticeable speed fluctuations, with the instantaneous estimation error occasionally exceeding ±100r/min. The proposed LADRC-PLL significantly attenuates these noise-induced oscillations, confining the speed error predominantly within ±40r/min. This noise suppression capability is quantitatively reflected by the RMSE, which drops from 22.6r/min with the PI-PLL to 6.9r/min with the LADRC-PLL.

The performance gap widens significantly when the noise amplitude is increased to 0.15A. The PI-PLL suffers from severe degradation, yielding highly oscillatory speed estimates with error spikes approaching ±400r/min, which risks destabilizing the closed-loop control. Conversely, the LADRC-PLL maintains a bounded error profile, effectively filtering out the severe noise. The corresponding RMSE is limited to 20.7r/min, which is less than one-third of the 68.9r/min produced by the PI-PLL. These results substantiate the superior high-frequency noise rejection capability of the adaptive-bandwidth observer under nonideal sensing conditions.

**Figure 10:**
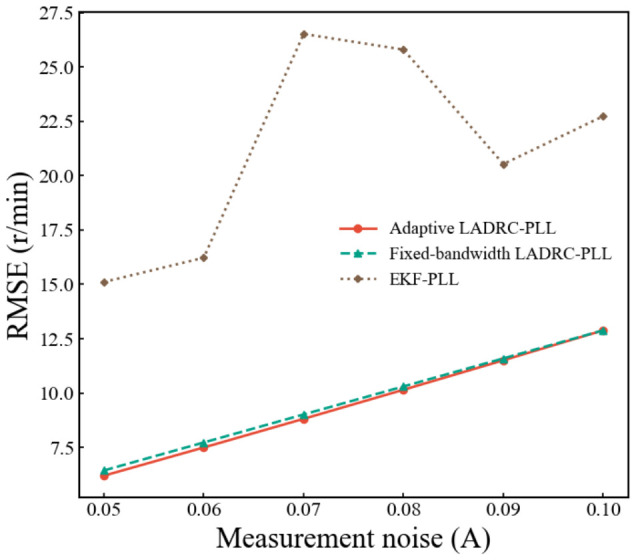
Speed estimation errors (RMSE) of the evaluated algorithms under different measurement noise levels.

Figure 10 shows the speed estimation errors of the evaluated algorithms under different measurement noise levels. As the noise amplitude increases from 0.05A to 0.10A, the traditional EKF-PLL yields the highest RMSE and fluctuates significantly, reflecting its limited noise-rejection robustness. The fixed-bandwidth LADRC-PLL provides a more stable baseline, with its error scaling linearly with the noise. The proposed adaptive LADRC-PLL maintains a lower error than the fixed-bandwidth version across the entire test range, indicating that the adaptive mechanism successfully improves the steady-state estimation accuracy.

#### 4.3.2. ADC Quantization Effects

To investigate the robustness against quantization noise, the phase-current feedback is processed through simulated 12-bit and 16-bit ADCs. The speed estimation performance of the proposed LADRC-PLL is then evaluated against the conventional PI-PLL at a steady-state speed of 200r/min.

**Figure 11:**
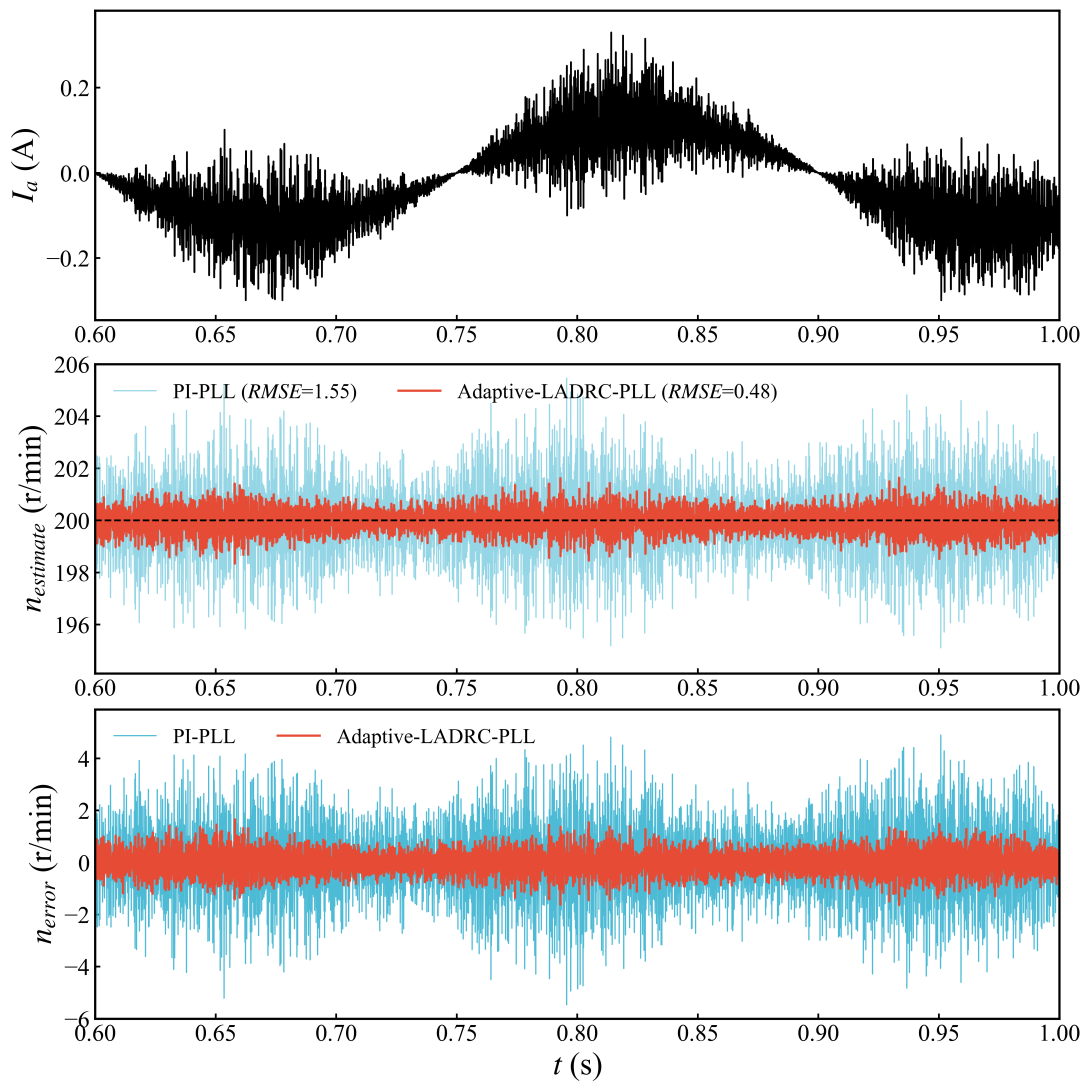
Speed estimation under 12-bit ADC quantization.

Under the coarse 12-bit ADC resolution (Figure 11), the quantized current induces pronounced chattering in the PI-PLL, resulting in a speed estimation ripple of approximately ±4r/min. The LADRC-PLL effectively absorbs these quantization harmonics, constraining the speed error predominantly within ±1r/min. Consequently, the RMSE drops from 1.55r/min for the PI-PLL to 0.48r/min for the LADRC-PLL.

**Figure 12:**
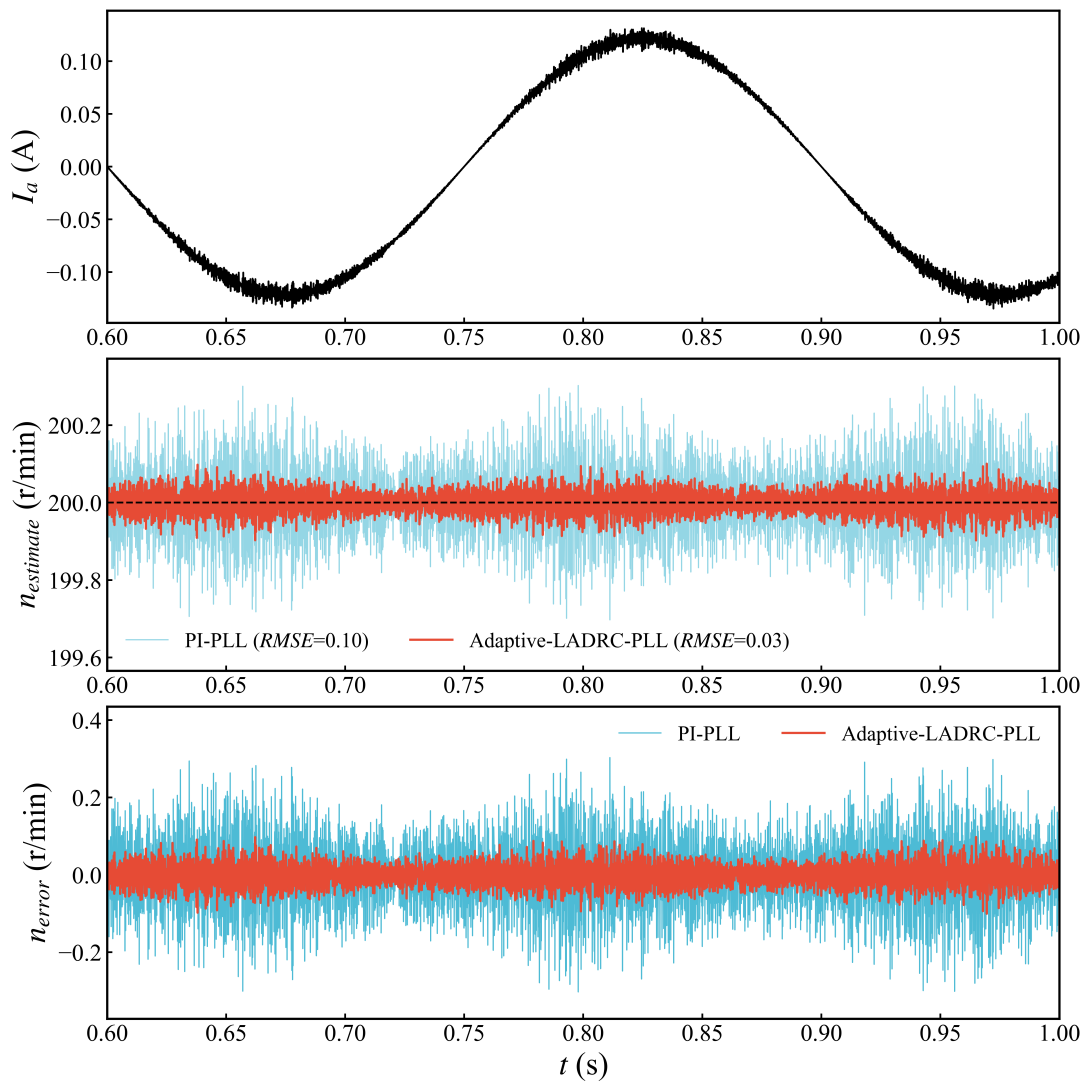
Speed estimation under 16-bit ADC quantization.

When the ADC resolution is increased to 16 bits (Figure 12), the finer quantization step inherently reduces the estimation variance for both algorithms. The PI-PLL limits the speed ripple to roughly ±0.2r/min. The LADRC-PLL further refines this trajectory, restricting the error band to an ultra-narrow ±0.05r/min. The corresponding RMSE decreases from 0.10r/min to 0.03r/min, confirming that the adaptive-bandwidth LESO consistently mitigates low-amplitude, high-frequency quantization disturbances.

**Figure 13:**
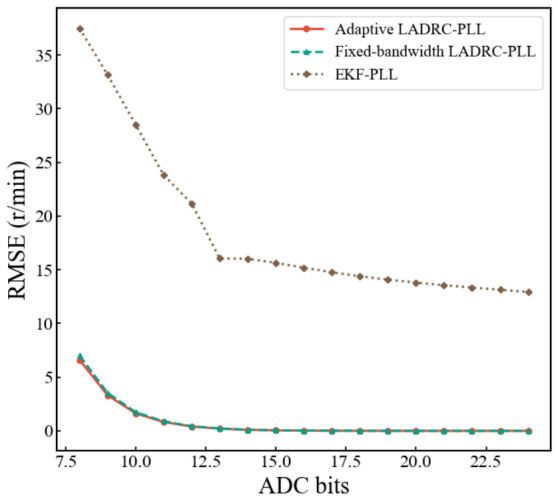
Speed estimation errors across different ADC resolutions.

Figure 13 illustrates the speed estimation errors across different ADC resolutions, where the traditional EKF-PLL yields the highest overall error and degrades sharply under severe quantization noise. Both LADRC methods demonstrate strong inherent robustness, with their errors rapidly decreasing to near-zero levels as the bit resolution increases. However, under extreme low-resolution conditions, the proposed adaptive LADRC-PLL still maintains a marginal but consistent precision advantage over the fixed-bandwidth version, confirming the adaptive mechanism’s capability to further suppress severe truncation errors.

#### 4.3.3. Current Sensor DC Offset

**Figure 14:**
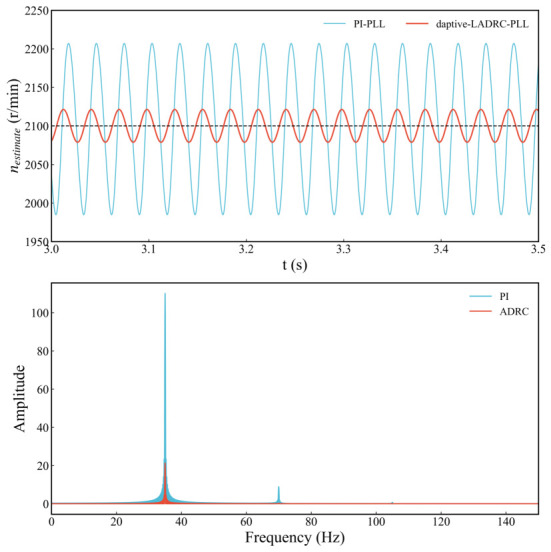
Effect of current sensor DC offset on speed estimation.

Current sensor DC offset introduces a persistent bias in the reconstructed current components, leading to a steady coordinate transformation error. This residual periodic disturbance manifests as steady-state speed ripple. The time-domain waveforms and the corresponding frequency spectra are evaluated in Figure 14.

Operating at a nominal speed of 2100r/min, the PI-PLL exhibits severe steady-state oscillations with a speed ripple approaching ±110r/min. The proposed LADRC-PLL effectively suppresses this disturbance, confining the speed ripple to approximately ±20r/min. In the frequency domain, the DC offset generates a dominant harmonic component at 35Hz. The PI-PLL yields a prominent spectral peak with an amplitude exceeding 100. For the LADRC-PLL, this corresponding peak is substantially attenuated to an amplitude of roughly 20. These results demonstrate that the LESO-based extended-state estimation successfully compensates for DC offset disturbances, ensuring tight speed stabilization under nonideal sensing conditions.

## 5. Conclusions

This paper presented an L-SHADE-optimized adaptive LADRC-PLL scheme to address the fundamental trade-off between dynamic tracking speed and noise immunity in sensorless PMSM drives. By modeling the electrical-angle mismatch as a lumped disturbance within a virtual coordinate frame, LESO dynamically adjusts its bandwidth according to real-time estimation errors. Extensive evaluations demonstrated that the proposed method ensures rapid transient accommodation with a small speed dip under abrupt load torque changes, while maintaining tight steady-state regulation under complex nonidealities. For a large speed command step, a short-lived speed overshoot may appear as a trade-off for aggressive tracking (see Section 4), yet the response remains well damped and settles quickly. Quantitatively, the adaptive mechanism significantly improves estimation accuracy over conventional PI-PLL designs. Under severe measurement noise of 0.15A, the speed estimation RMSE was reduced by approximately 70% (from 68.9 to 20.7). Similarly, under coarse 12-bit ADC resolution, the RMSE dropped from 1.55 to 0.48, and the dominant periodic perturbations induced by current sensor DC offsets were substantially attenuated.

These findings indicate that the integration of virtual-coordinate disturbance modeling and evolutionary-algorithm-optimized adaptive bandwidth tuning provides a highly robust sensorless control solution, making it highly suitable for industrial drives operating under strict noise, quantization, and sensor bias constraints. However, it should be noted that the current performance is evaluated under simulation environments, which may not fully capture unmodeled high-frequency inverter nonlinearities or extreme thermal variations present in physical systems.

Future work will focus on deploying the proposed control framework on a hardware testbench to characterize its stability margins and computational overhead in real-time digital signal processors. Further investigations will also explore compensating for inverter dead-time effects to extend the robust operational range of the sensorless drive into ultra-low-speed regions.

## Figures and Tables

**Table 1:** Main parameters of the PMSM.

Parameter	Symbol	Value	Unit
Stator resistance	$R_{s}$	5.3	Ω
Stator inductance	$L_{s}$	8.6	mH
PM flux linkage	$\psi_{f}$	0.28	Wb
Moment of inertia	$J_{m}$	0.008	kg·m^2^
Viscous friction coefficient	*B*	0.001	N·m·s/rad
Pole pairs	*p*	2	—
Rated speed	*n*	2000	r/min

## Data Availability

The raw data supporting the conclusions of this article will be made available by the authors on request.

## Nomenclature

*B*	Viscous friction coefficient (N·m·s/rad)
$J_{m}$	Rotor moment of inertia (kg·m^2^)
J(p)	L-SHADE fitness function used for adaptive-parameter optimization
$R_{s}$, $L_{d}$, $L_{q}$, $L_{s}$, $\psi_{f}$	Stator resistance (Ω), inductances (H), and PM flux linkage (Wb)
$i_{a,b,c}$, $i_{d,q}$, $i_{\gamma ,\delta }$	Phase, *d*-*q*, and virtual-frame stator currents (A)
$v_{d,q}$, $v_{\gamma ,\delta }$	*d*-*q* and virtual-frame stator voltages (V)
$\psi_{d,q}$	*d*- and *q*-axis stator flux linkages (Wb)
$\theta_{e}$, ${\hat{\theta }}_{e}$, $\Delta \theta_{e}$	Actual, estimated, and error electrical angles (rad)
$\omega_{e}$, ${\hat{\omega }}_{e}$	Actual and estimated electrical angular speeds (rad/s)
*n*, $n^{⋆}$	Mechanical speed and speed reference (r/min)
$T_{e}$, $T_{s}$, $M_{p}$	Electromagnetic torque (N·m), settling time (s), and peak overshoot (%)
$d_{\gamma ,\delta }\left(t\right)$, $d_{\gamma ,\delta }^{\theta }\left(t\right)$, $d_{\mathrm{unc},\gamma ,\delta }\left(t\right)$	Total, angle-error, and uncertainty disturbances (A/s)
$x_{1}$, $x_{2}$, *y*, *u*, $b_{0}$, f(t)	ADRC canonical states, output, input, input gain, and disturbance
$z_{1}$, $z_{2}$, $z_{3}$, $\beta_{1}$, $\beta_{2}$, $\beta_{3}$	LESO state estimates and observer gains
$\omega_{o}$, $\omega_{o}^{⋆}$, $\omega_{\min}$, $\omega_{\max}$	Actual, target, minimum, and maximum observer bandwidths (rad/s)
$e_{\mathrm{obs}}$, $k_{\omega }$	LESO innovation and bandwidth sensitivity coefficient
$\tau_{\omega }$	Smoothing time constant (s)
p, *D*, *H*, $NP_{G}$	Design vector, problem dimension, memory size, and population size
$x_{i,G}$, $v_{i,G}$, $u_{i,G}$	Target, mutant, and trial vectors in L-SHADE
$F_{i,G}$, $CR_{i,G}$, $M_{F}$, $M_{CR}$, $w_{i}$	L-SHADE scale factor, crossover rate, memories, and weight
*p*	Pole pairs or *p*-best proportion in L-SHADE
